# Case Report: Iatrogenic intraperitoneal hematoma compresses the ureters leading to urogenic sepsis

**DOI:** 10.3389/fmed.2025.1574196

**Published:** 2025-07-15

**Authors:** Zhengyi Zhang, Peng Ding, Meijie Yang, Xiujuan Zhou, Kunlan Long

**Affiliations:** ^1^School of Clinical Medicine, Chengdu University of Traditional Chinese Medicine, Chengdu, China; ^2^Department of Critical Care Medicine, Hospital of Chengdu University of Traditional Chinese Medicine, Chengdu, China

**Keywords:** sepsis, iatrogenic hematoma, urinary sepsis, compression, case report

## Abstract

**Background:**

Urogenic sepsis is a systemic inflammatory response syndrome triggered by infections of the genitourinary tract. It is characterized by complex etiology, atypical clinical symptoms, rapid disease progression, and difficulty in treatment, making it a focal point and challenge in clinical diagnosis and therapy. Although endovascular interventions are effective in treating conditions such as iliac artery thrombosis, they can also lead to complications such as arterial rupture and subsequent hematoma formation. This article reports a rare case of urogenic sepsis that developed secondary to urinary tract obstruction and infection caused by compression of the ureter due to an iatrogenic retroperitoneal hematoma following endovascular surgery.

**Case introduction:**

A 63-year-old male patient was admitted to the hospital with symptoms of severe viral pneumonia and acute heart failure. Two months earlier, the patient had undergone a vascular interventional procedure, during which a rupture of the left iliac artery led to the formation of an intra-abdominal hematoma, a condition that was not given due attention at the time. Although initial treatment was provided for the respiratory and cardiac issues, the patient’s condition continued to deteriorate. Subsequent examinations revealed that the hematoma was compressing the ureters, causing severe ureteral obstruction and urinary sepsis. The patient then underwent an emergency transurethral ureteral stent implantation. Following the procedure, the patient’s condition significantly improved, and he was eventually discharged from the hospital.

**Conclusion:**

Cases of iatrogenic intraperitoneal hematoma compressing the ureters and subsequently causing urogenic sepsis are extremely rare in clinical practice. This case highlights the necessity of early identification and intervention of iatrogenic complications, especially in the patient population with a history of vascular interventional surgery. It underscores the crucial role of targeted diagnostic strategies and surgical interventions in resolving the challenge of mechanical obstruction. The research findings indicate that it is essential to establish a multidisciplinary collaborative mechanism to effectively manage such complex cases.

## 1 Introduction

Sepsis refers to a dysregulated host response to infection that results in life-threatening organ dysfunction (1). Among them, septic shock, as a severe subtype of sepsis, is characterized by significant circulatory and cellular metabolic abnormalities, and its in-hospital mortality rate is as high as 40% (1). In clinical practice, prompt etiological identification and primary lesion control in patients with suspected sepsis or septic shock are key to treatment (2). Urinary sepsis refers specifically to sepsis caused by genitourinary tract infection (3). Common causes include upper urinary tract obstruction and genitourinary surgical procedures (4, 5). Flow-based studies have shown that ureteral stones and ureteral stricture deformities are the main causes of obstructive uropathy in middle-aged people (6–8). Of note, the prevalence of severe sepsis in general hospitals in patients with acquired urinary tract infection is 2%, while the prevalence of septic shock is only 0.3% (5).

In the field of vascular intervention, endovascular intervention has become the preferred treatment for intrailiac aortic thrombosis. However, the endovascular Surgery is associated with a variety of complications, including self-limited hematoma at the puncture site, dissection formation (involving the iliac artery and aorta), vascular perforation, distal embolization, and contrast-related adverse effects (9, 10). Arterial rupture is a rare but serious complication, with an incidence of about 0.5% ~ 3.0%, and it is necessary to urgently place a covering stent to control bleeding (11, 12). At present, there have been no case reports of urogenic sepsis caused by ureteral compression by iatrogenic abdominal hematoma. This article reports a rare case of urinary sepsis caused by hematoma compression of the ureter due to complications related to the operation during thrombectomy.

## 2 Case presentation

On December 22, 2024, a 63-year-old male patient was admitted to the Department of Cardiovascular Medicine of our hospital who complained of “dyspnea with cough and sputum for 3 days.” Three days ago, the patient developed an unexplained cough with thick yellow sputum and progressively worsening dyspnea. On admission, he was shown to be listless, orthopnea, coarse breath sounds in both lungs, and crackles in the middle and lower lung fields. Chest CT showed scattered infection foci in both lungs with a small amount of bilateral pleural effusion; Emergency ECG Graph shows atrial fibrillation with a rapid ventricular rate (160 beats/min). Symptomatic treatment was given with intravenous pumping of recombinant human brain natriuretic peptide and amiodarone, budesonide suspension and combined ipratropium bromide nebulized inhalation. With a transcutaneous oxygen saturation of 80% (mask oxygen) and a heart rate of up to 160 beats/min (pumped amiodarone), he was preliminarily diagnosed with “severe pneumonitis, acute heart failure, and acute respiratory failure,” and was transferred to the ICU for further treatment. Two months ago, the patient underwent “left lower extremity femoral artery tomy thrombectomy + left iliac artery balloon dilation,” and the left iliac artery ruptured during the operation, resulting in the formation of intra-abdominal hematoma (Precise implantation of a covered stent at the site of vascular rupture to repair the vessel wall. No treatment was performed for intra-abdominal hemorrhage).

After admission to the IC U, the physical examination showed that T: 36 0.5°C, HR: 144 times/min, invasive blood pressure: 143/71 mmHg, RR: 34 times/min. The extremities are clammy and cold, and the lower extremities are spotted. Ancillary examinations: blood gas analysis showed metabolic acidosis with hypoxemia (pH:7.28 [normal range 7.35–7.45], PO_2_:77.83 mmHg [normal range 83.0–108.0 mmHg], oxygenation index:155.66 mmHg), lactate level 7.35 mmol/L (normal range 1.0–1.8 mmol/L); Thirteen nucleic acid tests for respiratory pathogens showed positive nucleic acid for influenza A virus H1N1 (2009). The B-type natriuretic peptide precursor is 14811.7 pg/mL (reference range: 0-450 pg/mL). A routine echocardiogram shows a left ventricular ejection fraction of 51% (the measured value of left ventricular systolic function is at the lower limit of the normal range). Urinalysis showed no leukocyte esterase or nitrite. The preliminary diagnosis is:(1) Severe viral pneumonia; (2) Sepsis (lung infection); (3) Acute heart failure; (4) Acute respiratory distress syndrome. The patient received rapid fluid resuscitation, Milrinone enhances myocardial contractility, and furosemide reduces the afterload of the heart, piperacillin sodium tazobactam sodium combined with moxifloxacin anti-infection, oral oseltamivir antiviral, The patient was simultaneously receiving non-invasive mechanical ventilation (ventilator settings: Mode: P-SIMV, PS: 10 cmH_2_O, FiO_2_: 60%, PEEP: 5 cmH_2_O, RR: 17 breaths per minute, PC: 10 cmH_2_O). On December 25, 2024, due to hemodynamic instability and a persistent decline in oxygen saturation, the patient underwent bedside oral endotracheal intubation (invasive mechanical ventilation settings: Mode: P-SIMV, PS: 15 cmH_2_O, FiO_2_: 60%, PEEP: 12 cmH_2_O, RR: 20 breaths per minute, PC: 15 cmH_2_O) and combined circulatory support therapy (norepinephrine [0.88 μg/kg/min] + epinephrine [0.02 μg/kg/min] + milrinone [0.56 μg/kg/min]).

**Figure 1:**
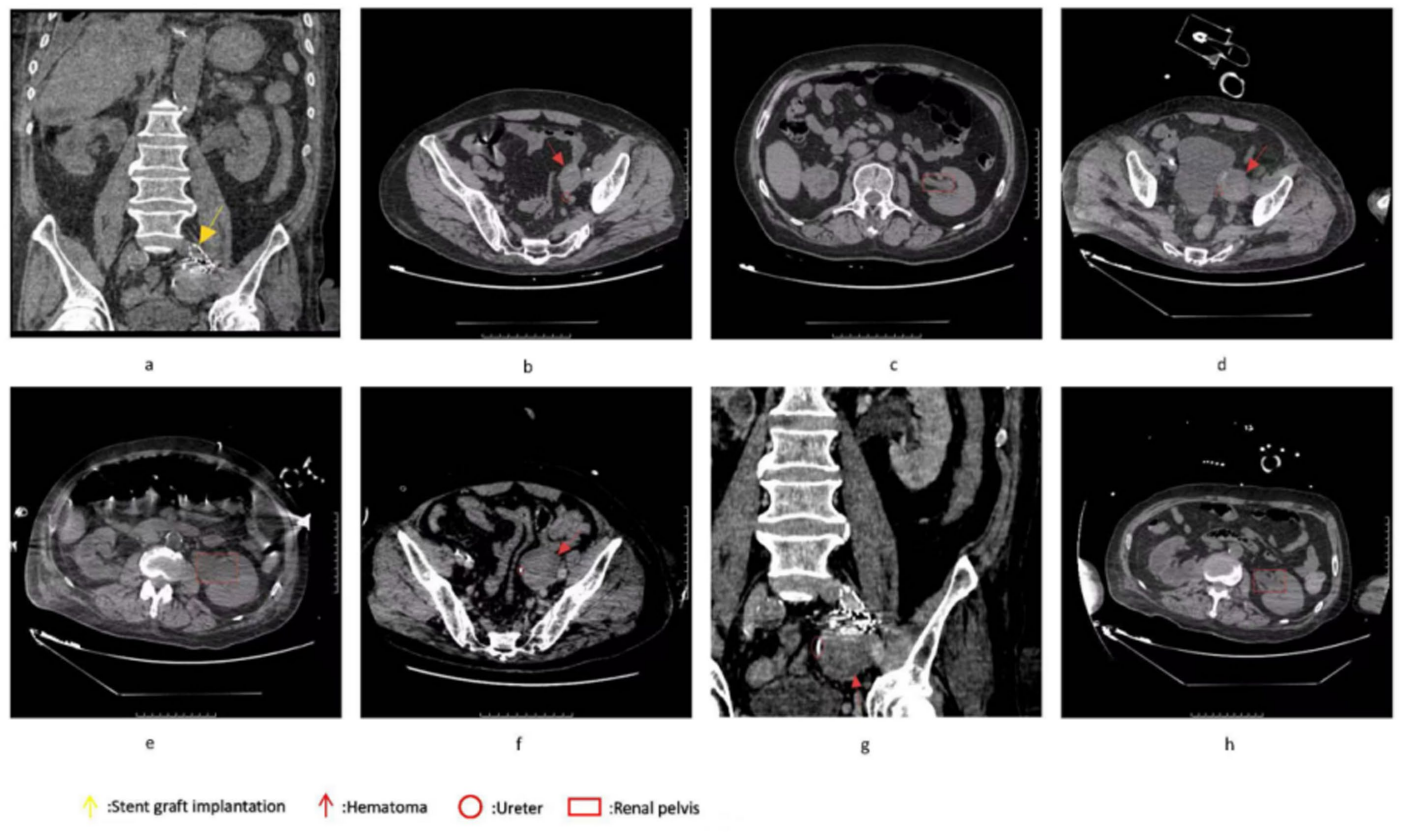
**(a)** shows the emergency implantation of a covered stent following an iliac artery rupture. **(b,c)** Depict the abdominal CT scans performed on October 23, 2024, as part of the follow-up after vascular interventional surgery. At this time, the hematoma measured 3.5 cm × 2.6 cm. Importantly, the hematoma did not compress the ureter, and there was no hydronephrosis observed in the left renal pelvis on December 25, 2024, the abdominal CT scans **(d,e)** revealed that the hematoma had increased in size to 4.6 cm × 4.5 cm. Additionally, the mid-portion of the left ureter exhibited tortuosity. Moreover, hydronephrosis and dilation of the upper and middle segments of the left ureter were noted. Subsequently, a ureteral stent was implanted, as illustrated in panel **(f,g)**. Following this intervention, a follow-up examination on January 3, 2025 **(h)** showed a significant alleviation of the hydronephrosis in the left renal pelvis.

The patient’s blood routine examination revealed significantly elevated inflammatory markers: the white blood cell count was 14.61 × 10^9^/L, with a neutrophil count of 13.66 × 10^9^/L. The level of C-reactive protein in whole blood was 24.44 mg/L, and procalcitonin (PCT) was 1.54 ng/mL. Additionally, the patient’s lactate level was elevated, exceeding 2 mmol/L, and required a large dose of norepinephrine to maintain blood pressure. Considering the clinical manifestations and laboratory findings, septic shock is highly suspected in this patient. To further clarify the diagnosis, immediate chest and abdominal CT scans were arranged for the patient. The CT results showed that: 1. Hydrops in the left renal pelvis and the middle and upper part of the left ureter were dilated, and the middle and upper part of the left ureter was tortuous; 2. The occupancy of the left side of the pelvic wall has been changed, showing a slightly higher density shadow in a similar circular shape, and the scope is about the same 4.6 cm × 4.5 cm, the density of the inner part is high, and combined with contrast-enhanced CT, it suggests a possible hematoma (Figure 1d). Further tracing the medical history, on October 22, 2024, the patient underwent “left lower extremity femoral artery incision and thrombectomy + left iliac artery balloon dilation” in the Department of Vascular Surgery of our hospital for “left lower extremity artery thrombosis,” during which the iliac artery ruptured, resulting in intraperitoneal hemorrhage, and urgently underwent “left iliac artery stent grafting” (Figure 1a). Postoperative abdominal CT showed hematoma formation (Figures 1b,c).

Therefore, 2 months ago, due to improper vascular interventional procedure, the iliac artery ruptured and hemorrhage, forming an abdominal hematoma, the hematoma compressed the left ureter, causing the ureter to travel and twist, the left renal pelvis and the upper end of the ureter to expand and accumulate hydrops, obstructive pyelonephritis (Figure 1e), and finally caused complicated urinary tract infection, and progressed to urogenic sepsis. The definitive diagnosis is: (1) obstructive pyelonephritis, (2) complicated urinary tract infection, (3) urogenic sepsis, (4) intra-abdominal hematoma compression of the ureters (iatrogenic). Contact the Department of Urology immediately for emergency “transurethral ureteral stent implantation.” The operation process is as follows: the patient takes the lithotomy position, after general anesthesia, uses the 8/9.8Fr ureteroscope to insert the ultra-slippery guidewire retrograde into the left ureter under direct vision (No thickening of the ureteral wall observed), and sees that the lumen of the lower part of the left ureteral is slightly narrow, and it is difficult to enter the lens, so the scope is withdrawn to the opening of the left ureter, and 1 5F double “J” tube is inserted into the ureter under the guidance of the ultra-slippery guidewire, and the catheter is successfully placed (Figures 1f,g). Anti-infective therapy was continued postoperatively. The patient’s infection indicators gradually decreased and the liver and kidney function returned to normal, which proved that the current treatment was effective. After 14 days of surgery, the patient recovered well and improved clinically, and was successfully discharged (Figure 1h).

## 3 Follow-up

**Figure 2:**
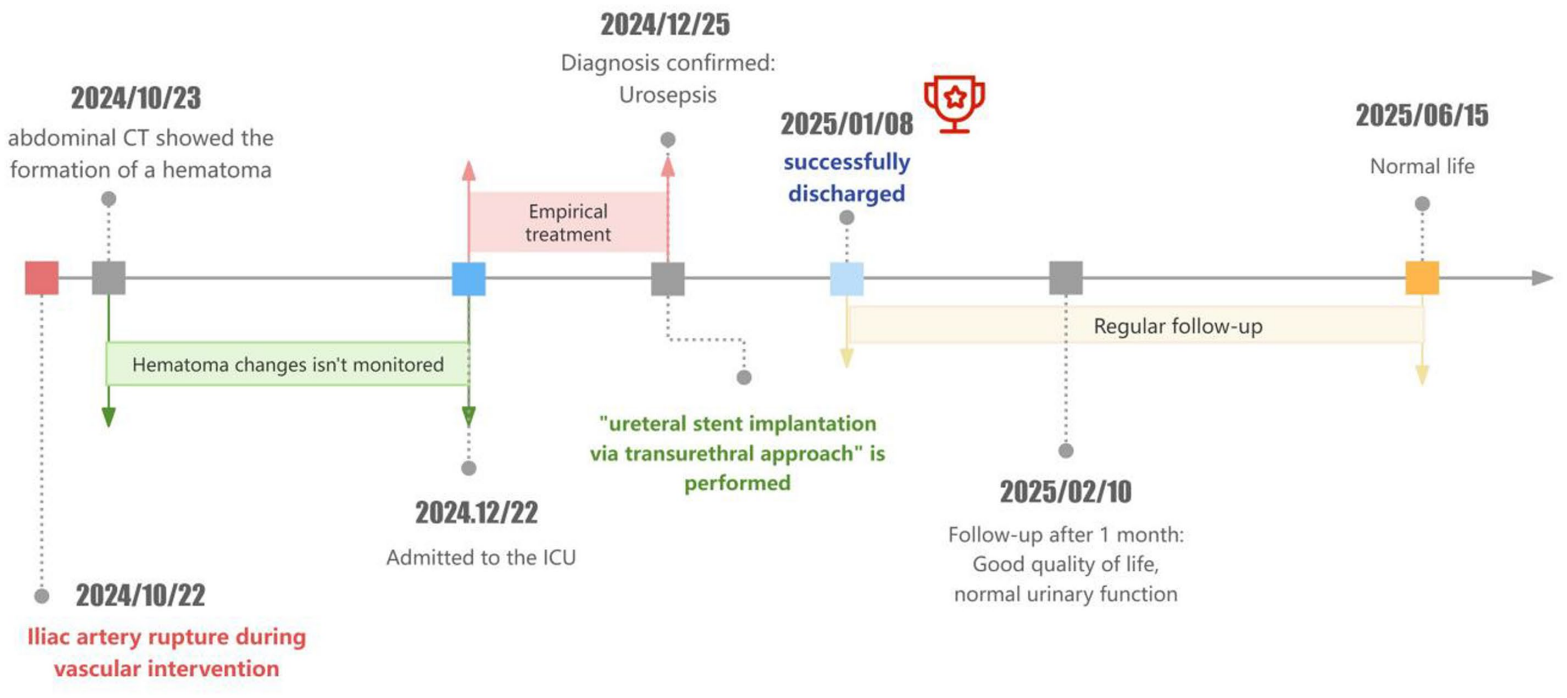
Diagnostic and treatment timeline.

After 1 months, the patient had a good quality of life, normal voiding function, and no other clinical symptoms (Figure 2).

## 4 Discussion and literature review

This section reports a clinical case of urinary sepsis caused by ureteral compression from an iatrogenic abdominal hematoma, which is a rare complication following endovascular intervention. Despite the absence of leukocyte esterase or nitrite positivity in the urine sediment test at admission, the patient was ultimately diagnosed with a urinary tract infection. This was because the hematoma compressed the upper segment of the ureter, confining the infection to the upper urinary tract without involving the bladder, thereby preventing the detection of lower urinary tract infection markers in the urine sediment test. Additionally, ureteral obstruction led to hydronephrosis, causing urine retention and inadequate concentration, which in turn resulted in low concentrations of inflammatory markers that were not detectable by routine tests. Finally, the patient was successfully relieved of ureteral obstruction and improved clinical prognosis by undergoing transurethral ureteral stent implantation.

Early recognition of sepsis and septic shock and prompt antibiotic intervention are key to reducing mortality (13). Appropriate initial antimicrobial therapy can significantly improve clinical outcomes in patients with sepsis (8, 14). However, the benefits and potential risks of early antimicrobial use need to be weighed against clinical practice, particularly in cases of sepsis due to noninfectious causes (15). It is worth noting that approximately 15 to 20% of sepsis cases are caused by noninfectious etiologies (16). Therefore, when clinical evidence does not support an infectious cause, the indiscriminate use of antimicrobial agents should be avoided, and reassessing the etiology is of paramount importance (2).

The clinical manifestations of urinary tract infections are significantly heterogeneous, ranging from asymptomatic bacteriuria to severe infections (17, 18). Although the overall incidence of sepsis is decreasing, the incidence of severe urinary tract infections continues to increase (3). Complicated urinary tract infections are often associated with host factors (eg, diabetes mellitus, immunosuppressive status) or anatomical/functional abnormalities of the urinary system (eg, urinary tract obstruction, detrusor dysfunction), which significantly increase the difficulty of infection control (19, 20). Urinary tract obstruction is one of the important causes of complicated urinary tract infections. Urinary tract obstruction can be classified into upper urinary tract obstruction and lower urinary tract obstruction based on the location of the blockage. Among these, unilateral ureteral obstruction is a common form of upper urinary tract obstruction, typically caused by ureteral stones, ureteral stricture, or tumor compression (21). It is extremely rare for a hematoma to compress the ureter as a result of iliac artery rupture caused by vascular surgery. Studies have shown that patients with upper urinary tract obstruction who do not receive prompt surgical decompression have a 29% increased mortality rate, and in-hospital mortality without surgery is more than twice that of the surgical group (22, 23).

Although endovascular intervention is an effective treatment for cardiovascular diseases, it inevitably causes damage to the blood vessel wall, thereby triggering a local inflammatory response and initiating the fibrosis process. Ureteral obstruction caused by fibrosis is the most common. During lower limb vascular surgery, the tissues surrounding the ureter may be implicated due to mechanical injury or local ischemia–reperfusion injury caused by surgical manipulation, thus triggering a local inflammatory response (24). The inflammatory response activates fibroblasts, prompting their transformation into myofibroblasts with synthetic capabilities (25). Myofibroblasts secrete a large amount of extracellular matrix components. Under normal physiological conditions, the synthesis and degradation of extracellular matrix components are in dynamic equilibrium. However, under inflammatory stimulation, the synthesis of extracellular matrix components increases while degradation decreases, leading to excessive deposition around the ureter (26). Meanwhile, surgical trauma and inflammatory response also lead to increased local oxidative stress levels. Excessive production of reactive oxygen species can damage cells and activate various signaling pathways, further promoting the expression of inflammatory factors and transforming growth factor β1, thereby exacerbating the fibrosis process (26). As fibrosis progresses, the scar tissue around the ureter gradually contracts, resulting in thickening of the ureteral wall and narrowing of the lumen. When the degree of narrowing reaches a certain level, it can cause ureteral obstruction, affecting the normal excretion of urine. The primary mechanism of ureteral obstruction caused by hematoma is the compressive effect on anatomical structures. Specifically, the hematoma directly compresses the ureter, causing narrowing of the ureteral lumen or even complete obstruction, thereby leading to impaired urine drainage.

It is worth noting that there is a significant temporal correlation between hematoma fibrosis and ureteral compression. In the later stages of hematoma, excessive fibrosis occurs. This fibrosis not only fills the hematoma cavity, but also directly compresses the ureter. Hematoma fibrosis is a complex and dynamic pathophysiological process. Its progression rate and severity are influenced by a combination of factors, including individual differences, the size and location of the hematoma, and the presence of infection. As fibrotic tissue proliferates and accumulates, its compressive effects on surrounding tissues and structures gradually become apparent. When this compression involves the ureter, it leads to entrapment and compression of the ureter, subsequently causing ureteral stenosis and, in severe cases, complete obstruction. Hematoma-induced extrinsic compression typically occurs in the early postoperative period (<3 months) (27). In this case, at 2 months postoperatively, the degree of hematoma fibrosis was sufficient to cause significant compression of the ureter, thereby leading to the emergence of relevant symptoms. In the early stages, fibrosis can be reversed through the body’s self-repair mechanisms. However, as damage accumulates, fibrous tissue gradually proliferates, arterial stiffness increases, and it may even spread to the surrounding interstitium, ultimately affecting the structure and function of the affected organs (28). Regardless of the etiology, ureteral obstruction induces morphological abnormalities in the renal parenchyma, especially the formation of renal fibrosis, by altering renal hemodynamics, impairing glomerular filtration function, and disrupting renal metabolism. Renal fibrosis is characterized by massive loss of parenchymal cells, excessive activation of fibroblasts, and deposition of fibrous connective tissue, ultimately leading to renal dysfunction or even renal failure (29, 30). It is worth noting that if urinary tract obstruction occurs after vascular intervention, damage to capillary endothelial cells, separation of pericytes from endothelial cells, rarefaction of capillaries, and reduced expression of both pro- and anti-angiogenic factors will accelerate glomerulosclerosis and renal tubulointerstitial fibrosis, making acute kidney injury or chronic kidney disease more likely to occur (31, 32). Chronic kidney disease (with a course exceeding 3 months) is characterized by persistent decline in renal function and structural changes in the kidneys. Renal fibrosis is the common pathway for its progression to end-stage renal disease and is also the core factor that endangers life. Therefore, how to effectively inhibit or reverse renal fibrosis has become the key to slowing the progression of chronic kidney disease and improving prognosis. Studies have shown that intervening in the transformation of pericytes into myofibroblasts and maintaining close contact between pericytes and endothelial cells may offer a new strategy for the prevention and treatment of renal fibrosis (33). However, currently there is a lack of clinically feasible methods to prevent the continued loss of renal function following the relief of ureteral obstruction. Delayed diagnosis and the absence of effective interventions after surgery remain significant causes of poor outcomes (34–36).

Fibrosis and hematoma compression are the two major pathological mechanisms of ureteral obstruction after endovascular surgery. Fibrosis is typically a chronic process, characterized on imaging by thickening of the ureteral wall and surrounding soft tissue density shadows; in contrast, hematoma compression is usually an acute process, characterized on imaging by the formation of a hematoma surrounding the ureter and its subsequent compression. The prognosis depends on the etiology of the ureteral obstruction and the timing of obstruction relief. Acute urinary tract obstruction is more likely to be reversible and less likely to cause damage to the renal filtration system or other functions. However, chronic obstruction can have more severe long-term effects on renal function (37). Unlike the acute presentation in our case, where the hematoma caused immediate compression and subsequent urosepsis, ureteral obstruction related to fibrosis typically presents with a gradual onset. In cases of ureteral obstruction caused by a hematoma, patients experience significant improvement in symptoms after the obstruction is relieved. However, in cases of ureteral obstruction caused by fibrosis, chronic renal damage persists even after the obstruction is resolved. In summary, while our case highlights the acute complications of iatrogenic peritoneal hematoma leading to ureteral obstruction and urosepsis, it is crucial to recognize that hematoma compression-induced obstruction is extremely rare and the primary mechanism underlying ureteral obstruction associated with endovascular surgery is fibrosis. Delayed infections after peripheral vascular stent surgery are rare, and the best practices for managing them are still unclear. The main goal of treatment is to completely eradicate the infection and restore blood circulation. Given this, we recommend that for any patient who shows early signs and symptoms of infection after vascular intervention, regardless of how long ago the surgery was, there should be a high level of vigilance for the possibility of delayed complications such as post-interventional infection. For patients with iliac artery rupture, clinical doctors should be alert to the possibility of urinary tract obstruction leading to urosepsis.

Based on the above evidence, the following points should be paid attention to in clinical practice: first, early identification of urinary tract obstruction is the key to effective intervention; Second, in patients with suspected urogenic sepsis, imaging should be the first diagnostic tool to determine whether there is an obstruction requiring urgent management; Finally, for the urine that is confirmed In cases of road infection and obstruction, early urological intervention is more clinically valuable than relying solely on advanced antibiotic therapy.

## 5 Research hotspots and frontiers

To achieve early and precise identification of ureteral compression and the resulting renal impairment, it is essential to widely implement novel imaging techniques and biomarker detection in clinical practice. On one hand, high-resolution multimodal imaging technologies should be utilized, such as the combination of contrast-enhanced CT and magnetic resonance urography, to more accurately identify minor compressive sources around the ureters. Moreover, real-time contrast-enhanced ultrasound can rapidly assess ureteral patency at the bedside, providing a more timely basis for clinical diagnosis. On the other hand, in terms of biomarker detection, the measurement of renal injury markers (such as NGAL and KIM-1) and fibrosis-related markers (such as TGF-*β*) should be promoted to enable early detection of renal impairment caused by ureteral compression and prompt intervention. For patients requiring relief of ureteral obstruction, the development of new types of ureteral stents is crucial. It is necessary to develop stents with antibacterial coatings to effectively reduce the risk of postoperative infections, or adjustable-length and shape stents that can better accommodate the anatomical structures of different patients, thereby enhancing the stability of the stents and patient comfort. These improvements will help enhance the efficacy and safety of ureteral stent implantation, leading to better patient outcomes.

## 6 Limitations

The patient visited the clinic due to upper respiratory symptoms. Based on the chest CT, echocardiogram, and laboratory tests, the preliminary diagnosis is severe viral pneumonia and acute heart failure. Although the patient had a recent (2 months ago) surgery, the initial diagnosis did not consider the possibility of urinary tract infection due to the lack of typical clinical manifestations (such as abdominal pain, change in urination habits, or decreased urine output) and significant symptoms of urinary tract infection. In the early stage of treatment, the patient was managed according to the routine protocols, without considering the possibility of postoperative complications. This diagnostic limitation leads to the failure of the initial treatment regimen to effectively control the source of infection, leading to disease progression. After conventional anti-infective therapy and fluid resuscitation failed to achieve the expected effect, we finally diagnosed urogenic sepsis through systematic review of medical history and improvement of relevant examinations, and adjusted the treatment regimen in time. It should be noted that this case report is based on a specific clinical situation, and its diagnosis and treatment process and results may be affected by individual differences and medical environment and other factors, so it is necessary to be cautious when generalizing to a wider patient population.

## 7 Conclusion

As a single-center case report, this study is based solely on the clinical data of a single patient. Individual differences among patients may significantly influence the clinical presentation, diagnosis, and treatment response of the disease. Therefore, the conclusions of this case report have obvious limitations when being generalized to a broader patient population. Although the ureteral stent implantation successfully relieved the urinary tract obstruction and significantly improved the prognosis in this case, this effect may not be applicable to all similar cases. To comprehensively evaluate the long-term efficacy and safety of ureteral stent implantation in iatrogenic urinary tract obstruction leading to urogenic sepsis, it is recommended to conduct multicenter cohort studies, enrolling more patients with different etiologies, disease courses, and treatment backgrounds, to validate its long-term efficacy and optimize the treatment protocols and clinical management strategies.

Persistent compression of the ureter by iatrogenic intra-abdominal hematoma is the key pathological mechanism leading to urinary tract obstruction, and the resulting urinary retention and dilation of hydrops in the middle and upper ureters constitute the main pathological basis for disease progression. Clinical observation has confirmed that timely surgical treatment of urology for such iatrogenic urinary tract obstruction can significantly improve the prognosis of patients. This finding has important clinical implications: (1) emphasizing the early identification and intervention of iatrogenic complications; (2) highlighting the importance of targeted diagnostic strategies; (3) to demonstrate the critical role of surgical intervention in the resolution of mechanical obstruction; (4) Suggest the necessity of establishing a multidisciplinary collaboration mechanism. Based on the experience of this case, it is recommended in clinical practice: (1) maintain a high degree of vigilance for patients with a history of iatrogenic procedures; (2) improve imaging examinations to identify urinary tract obstruction early; (3) Establish a rapid response mechanism and carry out surgical intervention in a timely manner; (4) Develop an individualized treatment plan.

## Data Availability

The original contributions presented in the study are included in the article/supplementary material, further inquiries can be directed to the corresponding author.
